# The Marine Microalga, *Tisochrysis lutea*, Protects against Metabolic Disorders Associated with Metabolic Syndrome and Obesity

**DOI:** 10.3390/nu13020430

**Published:** 2021-01-28

**Authors:** Claire Mayer, Léo Richard, Martine Côme, Lionel Ulmann, Hassan Nazih, Benoît Chénais, Khadija Ouguerram, Virginie Mimouni

**Affiliations:** 1 EA 2160 MMS, Mer Molécules Santé, IUML FR 3473 CNRS, Institut Universitaire Technologique, Département Génie Biologique, Le Mans Université, CEDEX 9, 53020 Laval, France; claire.mayer@univ-lemans.fr (C.M.); martine.come@univ-lemans.fr (M.C.); lionel.ulmann@univ-lemans.fr (L.U.); 2 GP Solutions, 98000 Monaco, Monaco; leo@gpsolutions.fr; 3 EA 2160 MMS, Mer Molécules Santé, IUML FR 3473 CNRS, UFR Pharmacie, Université de Nantes, CEDEX 1, 44035 Nantes, France; el-hassane.nazih@univ-nantes.fr; 4 EA 2160 MMS, Mer Molécules Santé, IUML FR 3473 CNRS, UFR Sciences et Techniques, Le Mans Université, CEDEX 9, 72085 Le Mans, France; benoit.chenais@univ-lemans.fr; 5 UMR1280 PhAN, Physiopathology of Nutritional Adaptations, INRAe, University of Nantes, CHU Hôtel Dieu, IMAD, CRNH Ouest, 44000 Nantes, France; khadija.ouguerram@univ-nantes.fr

**Keywords:** *Tisochrysis lutea*, DHA, obesity, metabolic syndrome, fat mass, glycemia, dyslipidemia, nonalcoholic fatty liver disease

## Abstract

Long-chain polyunsaturated fatty acids n-3 series and especially docosahexaenoic acid are known to exert preventive effects on metabolic disturbances associated with obesity and decrease cardiovascular disease risk. n-3 LC-PUFAs are mainly consumed in the form of fish oil, while other sources, such as certain microalgae, may contain a high content of these fatty acids. The aim of this study was to evaluate the effects of *Tisochrysis lutea* (Tiso), a microalga rich in DHA, on metabolic disorders associated with obesity. Three male Wistar rat groups were submitted for eight weeks to a standard diet or high-fat and high fructose diet (HF), supplemented or not with 12% of *T. lutea* (HF-Tiso). The supplementation did not affect plasma alanine aminotransferase (ALAT). Bodyweight, glycemia and insulinemia decreased in HF-Tiso rats (ANOVA, *p* < 0.001), while total plasma cholesterol, high-density lipoprotein-cholesterol (HDL-C) increased (ANOVA, *p* < 0.001) without change of low-density lipoprotein-cholesterol (LDL-C) and triacylglycerol (TAG) levels. Tiso supplementation decreased fat mass and leptinemia as well as liver TAG, cholesterol and plasma tumor necrosis factor-alpha levels (ANOVA, *p* < 0.001) while it did not affect interleukin 6 (IL-6), IL-4 and lipopolysaccharides levels. HF-Tiso rats showed an increase of IL-10 level in abdominal adipose tissue (ANOVA, *p* < 0.001). In conclusion, these results indicated that DHA-rich *T. lutea* might be beneficial for the prevention of obesity and improvement of lipid and glucose metabolism.

## 1. Introduction

The evolution of our society’s lifestyle, characterized by an increase in energy intake and a decrease in physical activity, is the main cause of the dramatic increase in the prevalence of overweight and obesity. Globally, the number of overweight and obese people tripled in 40 years, reaching more than 1.9 billion adults in 2016 [1]. Abdominal obesity is often associated with metabolic syndrome, a pathophysiological state defined by at least three of the following criteria: abdominal obesity, hyperglycemia, hypertriglyceridemia, and a decrease of plasma high-density lipoprotein-cholesterol (HDL-C) levels [2]. Inflammation, insulin resistance and an increase of plasma small, dense low-density lipoprotein-cholesterol (LDL-C) levels are often associated with metabolic syndrome and obesity [3]. These physiological and metabolic disturbances can lead to the development of nonalcoholic fatty liver disease (NAFLD), characterized by an excessive hepatic accumulation of triglycerides, more than 5.5% of liver weight [4,5].

Long-chain polyunsaturated fatty acids n-3 series (n-3 LC-PUFAs), particularly eicosapentaenoic acid (EPA) and docosahexaenoic acid (DHA) are known for their cardioprotective effects, as evidenced in numerous studies, notably for their antiatherosclerotic, antithrombotic and anti-arrhythmic effects [6,7,8]. Moreover, n-3 LC-PUFAs improve endothelial function and vasodilatation, decrease platelet aggregation, heart rate, blood pressure and the risk of ischemia and coronary artery disease [9]. The cardioprotective effects of n-3 LC-PUFAs are partly due to their preventive effects against dyslipidemia. Thus, n-3 LC-PUFAs exert hypotriglyceridemic effects that contribute to the decrease of plasma LDL-C and to the increase of plasma HDL-C levels [8]. n-3 LC-PUFAs can also inhibit crystalline cholesterol, thanks to their antioxidant capacities against reactive oxygen species (ROS) associated with cell membranes and lipoproteins [8].

n-3 LC-PUFAs exert anti-inflammatory effects by their ability to partially inhibit leukocyte chemotaxis, the expression of adhesion molecules and interactions between leukocytes and endothelial cells. n-3 LC-PUFAs decrease the production of pro-inflammatory eicosanoids (prostaglandins, leukotrienes) from arachidonic acid and decrease the production of pro-inflammatory cytokines through the inhibition of nuclear factor-kappa B (NF-κB). EPA and DHA can produce anti-inflammatory mediators such as resolvins, protectins and maresins [10]. n-3 LC-PUFAs also have the ability to interact with lipid rafts as mediators with anti-inflammatory properties [8].

n-3 LC-PUFAs have beneficial effects against NAFLD by decreasing lipid accumulation in the liver. In fact, n-3 LC-PUFAs inhibit hepatic lipogenesis by changing the expression and nuclear localization of sterol regulatory element-binding protein-1c (SREBP-1c), a transcription factor that controls several genes involved in lipogenesis, including acetyl-CoA carboxylase (ACC), fatty acid synthase (FAS) [8,11,12].

Moreover, in animal studies, n-3 LC-PUFAs were reported to improve insulin sensitivity despite the difficulty of demonstrating direct effects [8]. The increase of adiponectinemia induced by n-3 LC-PUFAs is correlated with an improvement of insulin sensitivity [8].

n-3 LC-PUFAs are highly present in fish such as salmon, herring or cod and are mainly commercialized as oils [13,14]. However, the decrease of fisheries resources related to marine pollution and overabundant fishing requires the search for new alternative sources rich in n-3 LC-PUFAs [13]. Thanks to their first place in the food chain, microalgae could be an interesting alternative to replace fish oils. In addition, they are less sensitive to heavy metal contamination [15]. Moreover, microalgae are a potential source of other highly bioactive molecules such as pigments, dietary fiber (soluble and insoluble), phytosterols or proteins [16]. These bioactive molecules are known to exert preventive effects against metabolic disturbances associated with fatness and would be an interesting alternative in the prevention of metabolic syndrome and obesity [17]. Nowadays, in the European Union (EU), the most popular microalgae widely commercialized as food ingredients are *Chlorella*, *Odontella aurita* and the procaryotic cyanobacterium *Arthrospira platensis*. Although they can be of interest to human health, other microalgae used in aquacultures, such as *Diacronema lutheri*, *Phaeodactylum tricornutum* and *Tisochrysis lutea*, are not yet considered as a food ingredient by the Novel Food Regulation of the EU [18].

*T. lutea* could be an interesting alternative to fish oil because of its many advantages: it has a rapid growth rate, a wide range of physicochemical tolerance and contains high levels of n-3 LC-PUFAs, especially DHA, as well as other bioactive molecules such as pigments (fucoxanthin, chlorophylls) phytosterols (brassicasterol, stigmasterol, fucosterol) and soluble fiber [19,20,21,22,23]. To our knowledge, no study has analyzed the effect of *T. lutea* as a dietary supplement against metabolic disorders associated with obesity.

The aim of this study was to evaluate the effects of the marine Haptophyte *T. lutea*, used as a food supplement, on metabolic syndrome associated metabolic disturbances. Thus, our work was to compare the effect of a high-fat (HF) diet associated with 10% of fructose in drinking tap water, known to induce obesity and metabolic syndrome [24,25], to that of the same HF diet supplemented with 12% of freeze-dried *T. lutea.* The animal model was a young male Wistar rat, which is commonly used to study metabolic syndrome and obesity. When this model is submitted to an HF diet for eight weeks, it adequately reproduces the different metabolic disturbances encountered in human disease [24,26,27,28]. Thus, in animal experimentations, over-consumption of fructose is involved in increasing body mass, energy intake, adiposity, glycemia, dyslipidemia and blood pressure [29]. The present study showed the preventive effects of *T. lutea* against metabolic disorders associated with metabolic syndrome and obesity, induced by HF and a high fructose diet.

## 2. Materials and Methods

### 2.1. Animal and Diets

To avoid age effects on metabolic disorders associated with obesity and any sexual endocrine fluctuation, eighteen male Wistar rats aged three weeks and weighing 130 ± 10 g were obtained from Janvier Labs (Le Genest Saint Isle, France). They were housed in a room under controlled conditions of temperature (22 ± 2 °C) and humidity (40–60%) and with a 12 h light/dark cycle. The rats were housed two per cage, 1291H Euro standard type III H in polycarbonate 425 × 266 × 185 mm (Tecniplast, Decines Charpieu, France). During one week of acclimatization, all animals were fed ad libitum with the standard diet A04 (SAFE, Augy, France) and with tap water. The nutritional protocol and all the experiments were approved by the Ethical Committee 06 Pays de la Loire and by the French Ministry of National Education, Higher Education and Research (APAFIS 10187, 31 August 2017).

Then, the animals were randomly divided into three groups of six rats and received diets ad libitum for eight weeks as follows: (1) the control (CTRL) group continued to receive the standard diet A04 providing 3.35 kcal/g, 72 kcal%, 19 kcal%, 8 kcal% from carbohydrates, proteins and lipids, respectively; (2) the HF group was fed the 260 HF diet (Safe, Augy, France) with 10% fructose in ad libitum drinking tap water (providing 1.67 kcal/mL) (Distriborg, St. Genis-Laval, France). HF diet provided 22 kcal/g, 61 kcal%, 24 kcal% from fat and carbohydrates, respectively; (3) the HF-Tiso group received an HF diet supplemented with 12% (*w/w*) of the freeze-dried microalga *T. lutea* (IBE-CNR, Florence, Italy). The dose of *T. lutea* supplementation was chosen from our previous studies that showed the beneficial effects of marine microalga *O. aurita* and *P. tricornutum* at 12% (*w/w*) after eight weeks of the diet [30,31]. These microalgae, which are rich in EPA, showed preventive effects against metabolic disturbances associated with obesity, induced by an obesogenic diet in Wistar rats [30,31]. Moreover, the selected dose of fructose supplementation was based on a meta-analysis highlighting that a dose of 10% fructose in drinking water was sufficient to induce the first characteristics of metabolic syndromes, such as an increase in body weight, blood pressure and glucose, insulin and triglyceride plasma levels in rats [24]. These data have been confirmed by the study of Toop and Gentili [32]. Freeze-dried *T. lutea* provided 5.27 kcal/g, 29 kcal%, 63 kcal%, 6 kcal% from crude protein, lipids and carbohydrates, respectively. Microalgal supplementation was incorporated directly in the HF diet to create a homogeneous mixture and provide 5.93 kcal/g in the HF-Tiso diet. DHA content in *T. lutea* was 1.41% of dry matter, equivalent to an average DHA intake of 20.3 mg/day/rat. HF diet was stored at +4 °C and *T. lutea* at −20 °C and renewed in the cages every three days for eight weeks.

The daily food and water consumption were evaluated in order to calculate energy intake. Energy intake (kcal/day) was calculated as the product of food consumption and dietary metabolizable energy. Food efficiency was estimated by using the following formula: (body weight gain (g)/energy intake (kcal)) × 100, adapted from Novelli et al. [33]. The bodyweight of the rats was monitored three times a week. Daily food and tap water consumption, and energy intake, were reported according to body weight.

The main characteristics and the fatty acid, pigment and sterol compositions of the CTRL diet, HF diet and *T. lutea* biomass are reported in Tables S1–S3.

The main components of the CTRL diet, HF diet and *T. lutea* are presented in Table S1. Data from CTRL and HF diets were provided by SAFE (Augy, France). *T. lutea* biomass was analyzed for proteins, carbohydrates, lipids, dietary fiber, ashes, and moistures. Total protein content was estimated as N × 6.25, where N is the nitrogen content determined through the elemental analysis. Carbohydrates were measured following Dubois et al. [34] and lipids following Marsh and Weinstein [35]. Total dietary fiber (TDF), insoluble and soluble dietary fiber (IDF and SDF) were determined by the AOAC Method 985.29 (AOAC Official Method 985.29) using commercial kits (Megazyme, Bray, Ireland). TDF was experimentally analyzed, not calculated as a sum of SDF and IDF. Moisture and ashes were determined following ISTISAN protocols (ISTISAN Report 1996/34, method B, p. 7; ISTISAN Report 1996/34, pp. 77–78, respectively). The fatty acid compositions of the CTRL diet, HF diet and *T. lutea* biomass are reported in Table S2. The determination of the fatty acid composition of the CTRL diet is described below. For the HF diet, the fatty acid composition was performed according to Simionato et al. [36]. The fatty acid analysis of *T. lutea* was determined according to the ISO 12966-4:2015 + ISO 12966-2:2011 procedures.

Pigment and sterol compositions, in vitro digestibility, and antioxidant activity of *T. lutea* are reported in Table S3. Pigment composition was determined by the SCOR-UNESCO method [37]. Carotenoid content was performed by HPLC analysis according to Van Heukelem and Thomas [38]. In vitro digestibility was evaluated by the method of Boisen and Fernández [39], modified as reported by Batista et al. [40].

Antioxidant activity of extracts in 90% acetone was measured by using the 2,2-diphenyl-1-picrylhydrazyle (DPPH) radical scavenger according to the method of Bondet et al. [41] with slight adaptations and reported in Table S3.

### 2.2. Blood and Organ Sampling

After the nutritional protocol, all rats were fasted for 12 h and anesthetized by intraperitoneal administration of a diazepam‒ketamine mix (4:3, *v/v*). Blood was collected from the abdominal aorta and sampled in coated tubes with 10% ethylenediaminetetraacetic acid (EDTA) from Sigma (St. Louis, MO, USA). Total blood was centrifuged at 1000× *g* for 10 min, the supernatant containing the plasma fraction was aliquoted in polyethylene tubes and stored at −20 °C. Liver, epididymal and abdominal adipose tissues were removed, rinsed with ice-cold NaCl solution (0.9%), weighed, frozen in liquid nitrogen, and stored at −80 °C until analysis.

### 2.3. Biochemical Analyses

Plasma levels of glucose, triacylglycerol (TAG), total cholesterol (TC), HDL-C, aspartate aminotransferase (ASAT) and alanine aminotransferase (ALAT) were measured by enzymatic methods using commercial enzyme kits (BIOLABO, Maizy, France). From ASAT and ALAT measures, ASAT/ALAT ratio was calculated. The atherogenic risk was evaluated by calculating the atherogenic index plasma (AIP) as the log (TAG/HDL-C) [42] and HDL-C/LDL-C ratio. Plasma LDL-C levels were estimated from the difference between TC and HDL-C. The insulin level was evaluated using an ELISA kit from Thermo Scientific (Waltham, MA, USA). The homeostasis model assessment of insulin resistance index (HOMA-IR) was estimated by calculating the fasting plasma glucose concentration (mg/dL) multiplied by fasting insulinemia (µUI/mL), divided by 405 [43]. Plasma pro-inflammatory cytokines, including interleukin-6 (IL-6), tumor necrosis factor-alpha (TNF-α), plasma and adipose anti-inflammatory cytokines such as interleukin-4 (IL-4) and interleukin-10 (IL-10), as well as plasma leptin, were quantified using rat enzyme-linked immunosorbent assay kits (ELISA) from Abcam (Cambridge, UK) according to the manufacturer’s protocols. The determination of serum endotoxin levels from Gram-negative bacteria was carried out with a commercial kit (Thermo Fisher, Waltham, Massachusetts, USA) using the Limulus amebocyte lysate (LAL) method and detected the serum LPS (lipopolysaccharide) level. This method is based on the proteolytic activation of proenzymatic factor C (present in circulating Limulus amebocytes) with endotoxins (LPS derived from the outer cell membrane of Gram-negative bacteria such as *E. coli*). The chromogenic assay of LAL determines endotoxin levels by measuring the activity of this protease in the presence of a synthetic peptide substrate that produces p-nitroaniline (pNA) after proteolysis. A yellow coloration was obtained, and absorbance was measured at 405 nm. Endotoxin levels were quantified from an endotoxin standard provided by the kit and derived from an *E. coli* O111:B4 strain. Results were expressed in EU (endotoxin unit)/mL.

### 2.4. Hepatic Lipid Measurements

TC and TAG levels were measured from an aliquot of liver total lipid extract by enzymatic methods using commercial enzyme kits (BIOLABO, Maizy, France).

### 2.5. Statistical Analysis

Data from experimental analyses are presented as mean values ± standard deviation (SD) (*n* = 6). After the analysis of variance by one-way ANOVA, the mean values were compared using Fisher’s least significant difference post hoc test (LSD). All statistical analyses were performed with Statgraphics Plus 5.1 (Manugistics Inc., Rockville, MD, USA).

## 3. Results

### 3.1. T. lutea Supplementation Decreases Body Weight and Fat Mass in Wistar Rats Fed a High-Fat Diet

#### 3.1.1. Nutritional Monitoring: Food and Water Intake

**Figure 1:**
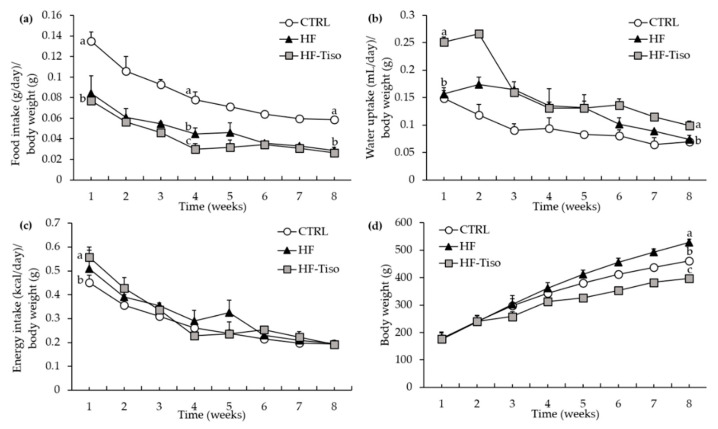
Effect of *T. lutea* supplementation on food intake (**a**), water intake (**b**), energy intake (**c**) and body weight (**d**) in HF-fed Wistar rats. CTRL (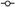), control group; HF (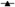), high-fat group; HF-Tiso (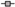), the high-fat group supplemented with *T. lutea*. Values are means (*n* = 6), with standard deviations represented by vertical bars. Statistical significance was determined using ANOVA with post hoc Fisher’s test, and means associated with letters indicate significative difference at *p* < 0.05 (week 1, Figure 1c) and *p* < 0.001 with a > b > c.

Food, water and energy intake were monitored for eight weeks (Figure 1a–c. The CTRL group displayed the highest food consumption/body weight ratio during the experimental period in comparison with the other groups (Figure 1a, ANOVA, *p* < 0.001). The HF and HF-Tiso groups presented similar food consumption/body weight ratios except for the fourth week, where HF rats exhibited a higher ratio than the HF-Tiso group (Figure 1a, ANOVA, *p* < 0.001). The ratio of water intake/body weight was markedly higher in the HF-Tiso group than other groups (Figure 1b, ANOVA, *p* < 0.001) except for the period between the third and fifth weeks, where no difference was observed between HF and HF-Tiso groups (Figure 1b). The ratio of water intake/body weight was similar between CTRL and HF rats throughout the protocol (Figure 1b).

#### 3.1.2. Energy Intake and Food Efficiency

Energy intake was calculated from water and food consumption, relative to body weight, and was similar between experimental groups except for the first week, where energy intake/body weight ratio was higher in the HF-Tiso group compared to the CTRL group (Figure 1c, *p* < 0.001). Food efficiency was calculated from body weight gain relative to energy intake and was similar between experimental groups, with the exception of the seventh week, where food efficiency was lower in CTRL rats compared to other groups (Figure S1, ANOVA, *p* < 0.01).

#### 3.1.3. Body Weight and Fat Mass

**Figure 2:**
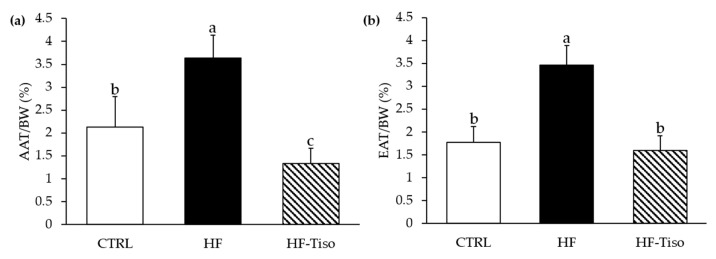
Effects of *T. lutea* supplementation on abdominal adipose tissue (**a**) and epididymal adipose tissue (**b**) weights. AAT, abdominal adipose tissue; CTRL, control group; EAT, epididymal adipose tissue; HF, high-fat group; HF-Tiso, the high-fat group supplemented with *T. lutea*. Values are means (*n* = 6), with standard deviations represented by vertical bars. Statistical significance was determined using ANOVA with post hoc Fisher’s test, and means associated with letters indicate significative difference at *p* < 0.001 with a > b > c.

Despite a ratio of energy intake/body weight similar between HF rats and those fed with *T. lutea* at the end of the nutritional protocol, the bodyweight of these two experimental groups was significantly different. Indeed, the final body weight was higher in the HF group compared to the other groups, and that of HF-Tiso rats was lower compared to other groups (Figure 1d, ANOVA, *p* < 0.001). In parallel, abdominal and epididymal adipose tissue weights increased with the HF diet compared with the other groups (Figure 2a,b, ANOVA, *p* < 0.001). Supplementation with *T. lutea* in HF rats significantly reduced abdominal as well as epididymal adipose tissue weight/body weight ratios compared to the HF group, while these ratios were similar between the HF-Tiso group and CTRL rats (Figure 2a, ANOVA, *p* < 0.001).

### 3.2. Effects of T. lutea Supplementation on Physiological and Metabolic Disorders in Wistar Rats Fed a High-Fat Diet

#### 3.2.1. Plasma Biochemical Parameters and HOMA-IR Index

In HF rats, it was observed an increase of ALAT plasma levels, associated with a decrease of plasma ASAT levels and ASAT/ALAT ratio, compared to CTRL rats (Table 1, ANOVA, *p* < 0.001). Plasma levels of ASAT were decreased in the HF-Tiso group, associated with a low ratio of ASAT/ALAT (Table 1, ANOVA, *p* < 0.001).

In the HF group, basal plasma levels of glucose, insulin and leptin were higher compared to CTRL and HF-Tiso rats (Table 1, ANOVA, *p* < 0.001). The supplementation with *T. lutea* partially prevented hyperinsulinemia observed in the HF group and restored the basal leptin level, whereas it was observed the lowest glycemia in the HF-Tiso group (Table 1, ANOVA, *p* < 0.001). In accordance with these results, the HOMA-IR index increased with the HF diet compared to CTRL rats, and it was decreased with *T. lutea* supplementation (Table 1, ANOVA, *p* < 0.001).

#### 3.2.2. Plasma Lipids, HDL/LDL Ratio and AIP Index

The HF group showed an increase in plasma TG, TC and LDL-C levels and a decrease in plasma HDL-C levels (Table 1, ANOVA, *p* < 0.001). *T. lutea* supplementation restored triglyceridemia and increased plasma HDL-C levels as well as plasma TC levels compared to CTRL and HF rats (Table 1, ANOVA, *p* < 0.001). LDL-C levels have not been restored after *T. lutea* supplementation (Table 1, ANOVA, *p* < 0.001). HF fed Wistar rats exhibited a high AIP index, an effective index associated with abdominal obesity, and a decrease in HDL/LDL ratio, an atherogenicity index (Table 1, ANOVA, *p* < 0.001), while *T. lutea* supplementation decreased the AIP restored HDL-C/LDL-C ratio (Table 1, ANOVA, *p* < 0.001).

### 3.3. Effects of T. lutea on Inflammatory Status

#### 3.3.1. Pro-Inflammatory and Anti-Inflammatory Cytokines

**Figure 3:**
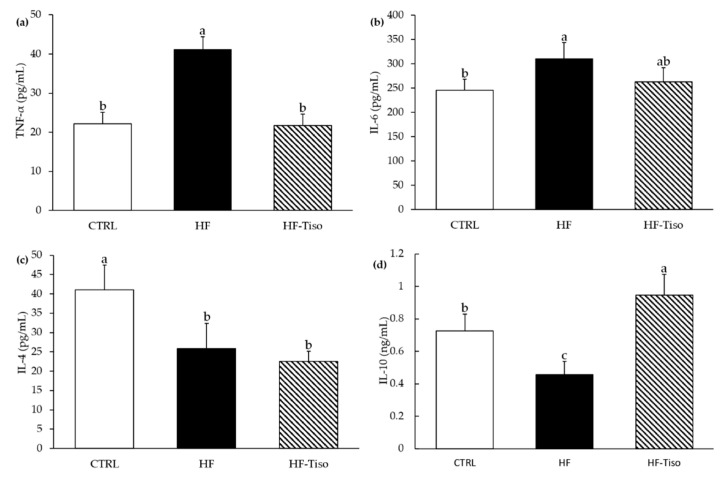
Effects of *T. lutea* supplementation on inflammatory and anti-inflammatory biomarkers. The plasma concentrations of (**a**) TNF-α, (**b**) IL-6, (**c**) IL-4 and adipocyte concentration in (**d**) IL-10. CTRL, control group; HF, high-fat group; HF-Tiso, the high-fat group supplemented with *T. lutea*; IL-4, interleukin 4; IL-6, interleukin 6; IL-10, interleukin 10; TNF-α, tumor necrosis factor-α. Values are means (*n* = 6), with standard deviations represented by vertical bars. Statistical significance was determined using ANOVA with post hoc Fisher’s test, and means associated with letters indicate significative difference at *p* < 0.001 with a > b > c.

As shown in Figure 3, plasma concentrations of pro-inflammatory cytokines, including TNF-α and IL-6, were significantly increased in the HF group compared to those in the CTRL and HF-Tiso groups (ANOVA, *p* < 0.001). The results also evidenced that *T. lutea* supplementation restored TNF-α concentration (ANOVA, *p* < 0.001), while plasma IL-6 concentration was not significantly decreased compared to the HF group. Anti-inflammatory cytokines were investigated in plasma and abdominal adipose tissue. The adipose tissue IL-10 concentrations and plasma levels of IL-4 were decreased with the HF diet compared to the CTRL diet (Figure 3, ANOVA, *p* < 0.001). Supplementation with *T. lutea* significantly improved inflammatory status by an increase of IL-10 levels in the adipose tissue, compared to the HF group (Figure 3, ANOVA, *p* < 0.001). Nevertheless, the plasma concentration of IL-4 in the HF-Tiso group was not restored compared to CTRL rats (Figure 3, ANOVA, *p* < 0.001).

#### 3.3.2. Serum LPS Levels

**Figure 4:**
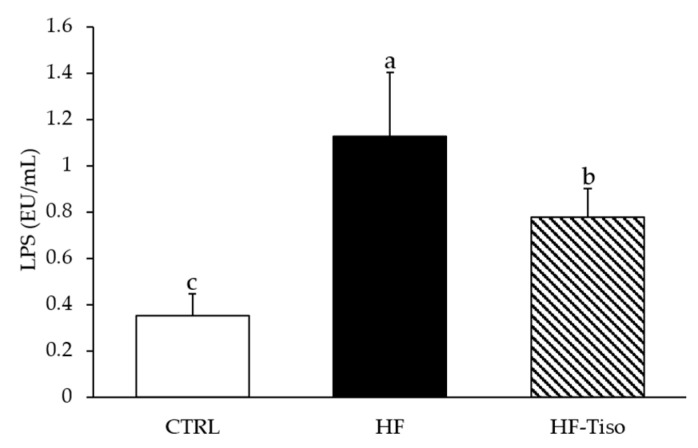
Effects of *T. lutea* supplementation on endotoxemia in HF-fed Wistar rats. CTRL, control group; HF, high-fat group; HF-Tiso, the high-fat group supplemented with *T. lutea*; LPS, lipopolysaccharide. Values are means (*n* = 6), with standard deviations represented by vertical bars. Statistical significance was determined using ANOVA with post hoc Fisher’s test, and means associated with letters indicate significative difference at *p* < 0.001 with a > b > c.

Serum LPS concentrations were measured in the different experimental groups (Figure 4). An increase of the LPS concentration in serum, named endotoxemia, was observed in Wistar rats fed the HF diet compared to those fed the standard diet (Figure 4, ANOVA, *p* < 0.001). The HF-Tiso diet improved serum levels of LPS compared to the HF group (Figure 4, ANOVA, *p* < 0.001).

### 3.4. Effects of T. lutea on Liver Triglyceride and Total Cholesterol Levels

**Figure 5:**
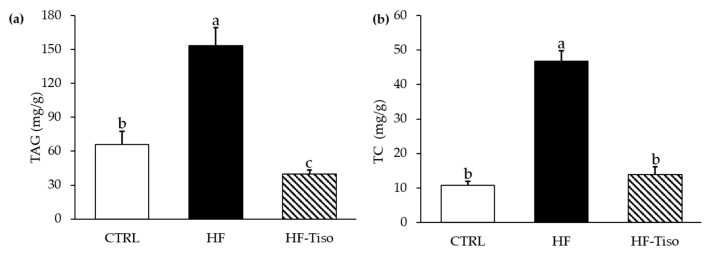
Effects of *T. lutea* supplementation on liver triglyceride (**a**) and cholesterol (**b**) levels. CTRL, control group; HF, high-fat group; HF-Tiso, the high-fat group supplemented with *T. lutea*; TAG, triacylglycerols; TC, total cholesterol. Values are means (*n* = 6), with standard deviations represented by vertical bars. Statistical significance was determined using ANOVA with post hoc Fisher’s test, and means associated with letters indicate significative difference at *p* < 0.001 with a > b > c.

The HF diet induced an increase in hepatic triglyceride and TC contents (Figure 5a,b, ANOVA, *p* < 0.001). *T. lutea* supplementation markedly decreased liver levels of triglycerides in HF-Tiso rats compared to the other experimental groups (Figure 5a, ANOVA, *p* < 0.001). In addition, hepatic total cholesterol levels were restored in the HF-Tiso group (Figure 5b).

## 4. Discussion

The aim of this study was to assess the impact of *T. lutea* used as a food supplement on metabolic disorders associated with metabolic syndrome and obesity, including overweight, dyslipidemia, inflammation and NAFLD. The results showed that *T. lutea* supplementation induced body weight and adipose tissue weight reduction. It also contributed to an improvement of plasma lipid parameters, insulinemia, leptinemia and inflammatory status and thus decreased atherogenic risk.

In accordance with the literature [24,26,27,28], our study highlighted that rats submitted to HF diet associated with fructose supplementation in drinking water (10%) presented increased body weight, fat mass and liver weight associated with high plasma levels of ALAT, a low ASAT/ALAT ratio and the presence of NAFLD. In addition, dyslipidemia was observed in association with a high AIP index and a low HDL/LDL ratio. Plasma levels of glucose, insulin, leptin, inflammatory cytokines, serum levels of LPS and HOMA-IR index were increased while anti-inflammatory cytokines were decreased.

### 4.1. T. lutea Supplementation Reduced Body Weight, Abdominal and Epididymal Adipose Weights in HF-Fed Wistar Rats

In the present study, a similar food and energy intake was observed between the HF and HF-Tiso groups, with the exception of the fourth week of the protocol. In parallel, the bodyweight of HF-Tiso rats was significantly reduced compared to the other experimental groups. This suggests that the decreased body weight observed in HF-Tiso rats is not caused by a deficit in energy intake and/or a decrease in food intake but may be due to the nutritional quality provided by *T. lutea* biomass and the potential synergistic effect of its biomolecules.

The high levels in DHA in *T. lutea* biomass (1.41% of dry weight, equivalent to 19.1 mg DHA/rat/day for the HF-Tiso group) could explain the reduction of body weight and fat mass observed in the HF-Tiso group. Indeed, previous studies conducted in animals showed the beneficial effects of DHA in reducing hypertrophy and hyperplasia of fat cells by activation of transcription factors involved in pre-adipocyte differentiation such as peroxisome proliferator-activated receptor-gamma (PPARγ) and the inhibition of mitogen-activated protein kinases (MAPK), involved in the last phase of adipocyte differentiation [44,45,46]. The study of Kim et al. [47] showed that DHA induces apoptosis of adipocytes before the differentiation stage and reduces lipid accumulation in adipocytes and the number of lipid droplets, through increased lipolysis associated with activation of PPARα as well as induction of uncoupling protein-2 (UCP2).

Furthermore, high levels of fucoxanthin in *T. lutea* biomass (64.2% of carotenoids, 9% of total pigments and 0.48% of dry weight, equivalent to 7 mg fucoxanthin/rat/day for the HF-Tiso group) could be another explanation for the decreased body weight and fat mass observed in HF-Tiso rats. In accordance with the present study, similar effects were observed in mice supplemented with a lipid fraction rich in fucoxanthin (9.6% of dry weight) from the macroalga *Undaria pinnatifida* [48]. Another study demonstrated that fucoxanthin could stimulate energy expenditures and β-oxidation, leading to a loss of body weight and fat mass [49]. Thus, it was shown that supplementation with synthetic fucoxanthin or *Undaria pinnatifida*-derived fucoxanthin (0.98 mg/g dry weight) at 400 mg/kg body weight in HF-fed rats, increased energy expenditures and β-oxidation, associated with decreasing gene expression involved in adipogenesis such as PPAR-α, peroxisome proliferator-activated receptor-gamma coactivator-1 alpha (PGC-1α), PPAR-γ and UCP1 [49].

A previous animal study demonstrated that chlorophyll supplementation (0.18 mg/10 g body weight/day) could also exert beneficial effects against obesity by the decrease of body weight gain, the improvement of glucose tolerance and the reduction of low-grade inflammation in HFD-fed C57BL/6 J male mice [50]. These effects could be due to the preventive action of chlorophyll supplementation on gut dysbiosis, characterized by the decreased Firmicutes/Bacteroidetes ratios, the increased abundance of Blautia bacteria, and the significant decrease of Lactococcus and Lactobacillus bacteria [50].

According to the study of Jung et al. [51], it was demonstrated that fucosterol, a phytosterol from the macroalga *Ecklonia stolonifera*, decreased the accumulation of 3T3-L1 pre-adipocytes via the inhibition of transcription factors PPARγ and CCAAT/enhancer-binding protein alpha (C/EBPα) [51]. Thus, fucosterol, abundantly found in *T. lutea* biomass (16.5% of total sterols, 0.23% of dry weight), could exert anti-obesogenic effects in rats supplemented with *T. lutea*.

Dietary fiber has positive effects for reducing body weight because it has decreased gastric emptying, slow energy and nutrient absorption, and may influence oxidation and storage of fat [52]. Their anti-obesogenic potential cannot be excluded and would be due to increasing intraluminal viscosity and fermentation of short-chain fatty acids [52]. These physiological changes would promote satiation and/or satiety and decrease food intake, as observed in rats supplemented by *T. lutea* in the fourth week of the protocol.

Furthermore, we can suggest that food intake/body weight ratio and indirectly the body weight and fat mass could be influenced by bioactive molecules like microalgal polysaccharides. Indeed, the effects of microalgal polysaccharides on intestinal microbiota are different depending on their nature and influence the regulation of appetite and weight gain. For example, it has been previously suggested that polysaccharides from the microalga *Isochrysis galbana* exert prebiotic effects through the beneficial increase of *Lactobacillus lactis* bacteria activity and reducing the growth and activity of enterobacteria and pathogens in diabetic rats supplemented with *Isochrysis galbana*, at a dose of 50 mg/day [53].

### 4.2. Effects of T. lutea, as a Dietary Supplement, in the Prevention of Dyslipidemia and Atherosclerosis

In the present study, *T. lutea* showed anti-dyslipidemic effects when used as a dietary supplement and could be explained by its high levels in bioactive molecules such as n-3 LC-PUFA, dietary fiber, phytosterols or fucoxanthin, which have beneficial effects in the regulation of lipid metabolism [54,55,56]. Fucosterol is also abundantly found in *T. lutea* biomass and showed the ability to increase plasma HDL-C levels [57].

Our results suggest the beneficial effects of soluble fiber (equivalent to 43 mg/rat/day for HF-Tiso rats) and DHA from *T. lutea* in reducing dyslipidemia. Indeed, dietary fiber is known for its beneficial effects on intestinal transit, thus playing a preventive role against cardiovascular diseases [58,59]. Moreover, hypotriglyceridemic properties of DHA have been shown in the literature and could be explained by the inhibition of triglyceride production and hepatic lipogenesis [8,60].

Dyslipidemia is a major risk factor in the development of atherosclerosis, and high AIP index and HDL/LDL ratio are, respectively, efficient markers of atherosclerosis and cardiovascular diseases [61,62,63]. In our study, rats supplemented with *T. lutea* showed a low AIP index compared to the other groups. In parallel, the HDL/LDL ratio was restored in HF-Tiso rats. These findings could be explained by the various molecules present in *T. lutea*, such as n-3 LC-PUFA, phytosterols and fiber, known to exert cardioprotective effects [64,65,66,67].

### 4.3. Effects of T. lutea Supplementation on Inflammatory Status in HF-Fed Wistar Rats

Decreased plasma levels of TNF-α pro-inflammatory cytokines have been observed in HF-Tiso rats. In parallel, although plasma levels of IL-6 and IL-4 were not restored, IL-10 level in abdominal adipose tissue was increased in the HF-Tiso group, reflecting the partial restoration of basal inflammatory status in rats supplemented with *T. lutea*. Our data may suggest that DHA from *T. lutea*, known for its anti-inflammatory effects, reduced production of pro-inflammatory cytokines and increased anti-inflammatory cytokine production [68]. Furthermore, similar effects were observed in a previous animal study that used DHA-rich oil from the microalga *Schizochytrium* sp. as a dietary supplement in C57BL/6 mice submitted to HF diet [69].

Fucosterol is known to have anti-inflammatory properties, mainly related to the inhibition of the nuclear factor-kappa B (NF-*κ*B) and p38 mitogen-activated protein kinases (p38 MAPK) pro-inflammatory pathways [70]. In addition, *T. lutea* is a significant source of anti-inflammatory compounds, including carotenoids [71]. As shown previously, fucoxanthin, at a dose of 0.6%, decreased the production of pro-inflammatory markers such as TNF-α and cyclooxygenase-2 (COX-2) in mice submitted to HF diet for four weeks [72]. In the present study, decreased inflammation in the HF-Tiso group could be explained by the synergistic effects of various bioactive molecules, including n-3 LC-PUFA and pigments.

In order to better understand the anti-inflammatory effects of *T. lutea* supplementation, serum LPS level was quantified in HF-Tiso rats. In agreement with the literature, the HF diet induced an increase of serum LPS level, defined as metabolic endotoxemia, by its capacity to modulate the intestinal microbiota. This change leads to an increase of intestinal permeability and then to the passage of LPS into the bloodstream [73]. Subsequently, the binding of LPS to adipocyte Toll-like receptor 4 (TLR4) receptors and pattern recognition receptors (PRRs) activates pro-inflammatory pathways such as Nf-κB, inducing pro-inflammatory cytokine production from adipose tissue [74]. By contrast, serum LPS level decreased in the HF-Tiso group, suggesting a preventive effect of *T. lutea* against metabolic endotoxemia. These observations could be attributed to n-3 LC-PUFA from *T. lutea* supplementation. Indeed, n-3 LC-PUFA demonstrated preventive effects against metabolic endotoxemia by their protective role against intestinal dysbiosis and permeability [73].

In accordance with our results, inflammation leads to hyperleptinemia, a marker of pro-inflammatory state and positively correlated with fat mass [74,75]. *T. lutea* supplementation, rich in DHA, restored plasma leptin level in HF-fed Wistar rats. The study conducted by Yook et al. [76] showed a leptinemia decrease after eight weeks of treatment in HF-fed C57BL/6 J mice supplemented with *Aurantiochytrium* microalga oil rich in DHA.

Fucoxanthin could be involved in leptinemia regulation in HF-Tiso rats. Indeed, a previous study showed the decrease of leptinemia in C57BL/6 J mice submitted for eight weeks to HF diet combined with *P. tricornutum* extract rich in fucoxanthin (corresponding to 0.2% fucoxanthin) [77].

### 4.4. Hypoglycemic and Hypoinsulinemic Effects of T. lutea Supplementation in HF-Fed Wistar Rats

Glycemia was significantly lower in HF-Tiso rats compared to the other groups. In parallel, plasma insulin level and HOMA-IR index were improved in the HF-Tiso group, suggesting a protective effect of *T. lutea* against insulin resistance. As well as other microalga species, *T. lutea* could have an anti-diabetic activity due to its high levels of bioactive molecules such as DHA and fucoxanthin [17,53,78,79,80]. Thus, the study of Yook et al. [76] showed preventive effects of *Aurantiochytrium* sp. microalga oil (rich in n-3 PUFA, DHA) against hyperinsulinemia in C57BL/6 J mice submitted to a hyperlipidic diet, suggesting the beneficial effects of n-3 LC-PUFA, and particularly DHA, in the improvement of insulin sensitivity. Indeed, a recent study showed that DHA significantly inhibits protein expression of the mechanistic target of rapamycin complex 1 (mTORC1) signaling pathway and increases phosphorylated-AKT protein (p-AKT) expression to reduce insulin resistance [81].

In another study, a lipid extract rich in fucoxanthin from the macroalga *Undaria pinnatifida* showed beneficial effects in the reduction of glycemia insulinemia in obese and diabetic mice [82]. This study suggests that fucoxanthin could improve insulin sensitivity and carbohydrate homeostasis through the regulation of glucose transporter-4 (GLUT-4), the reduction of hyperinsulinemia and neoglucogenesis, and the modification of the enzymatic activity of liver glucose regulatory enzymes such as glucose-6-phosphatase and phosphoenolpyruvate carboxykinase [83].

### 4.5. Effects of T. lutea on Non-Alcoholic Fatty Liver Disease

An excess of liver TAG and cholesterol levels can be hepatotoxic [84]. Thus, the integrity and metabolic functions of the liver were studied. In HF rats, the plasma ALAT level was increased and associated with a decrease of the ASAT/ALAT ratio, an indicator of hepatotoxicity in NAFLD [85]. Although ASAT/ALAT ratio was significantly lower in the HF-Tiso group due to low plasma ASAT levels, *T. lutea* supplementation did not impact plasma ALAT level and hepatomegaly induced by the HF diet, suggesting an absence of hepatotoxicity.

In parallel, *T. lutea* supplementation improved liver total cholesterol levels and significantly decreased TAG levels, suggesting NAFLD preventive effects of *T. lutea*. Similar effects were observed in Wistar rats supplemented for 66 days with freeze-dried *Diacronema vlkianum*, a marine microalga (equivalent to 101 mg/kg EPA and DHA in the diet), suggesting the preventive effect of n-3 LC-PUFA in the development of NAFLD [86]. DHA has been shown to inhibit lipid accumulation, particularly triglycerides, through the activation of free fatty acid 4 (FFA4) membrane receptor. Its activation prevents hepatic steatosis by inhibiting gene and protein expression of SREBP-1c through signaling cascade activation that involves Gq/11, calcium/calmodulin-dependent protein kinase kinase 2 (CaMKK) and AMP-activated protein kinase (AMPK) [87].

In addition, a protective effect of fucoxanthin from dried *U. pinnatifida* brown algae (0.2% fucoxanthin in diet) against liver lipid accumulation was demonstrated in C57BL/6 mice submitted to an HF diet through the reduction of activity of lipogenic hepatic enzymes such as glucose-6-phosphate dehydrogenase (G6PD), FAS and phosphatidate phosphatase (PAP), as well as the stimulation of enzymes involved in β-oxidation such as carnitine palmitoyltransferase 1a (CPT1a) [88]. Fucoxanthin also regulates the expression of genes associated with lipid metabolism, such as ACC, FAS and acyl-CoA cholesterol acyltransferase (ACAT), an enzyme that converts free cholesterol into cholesterol ester [89,90,91,92]. In parallel, fucoxanthin increases lipolysis through increasing expression of adipose triglyceride lipase (AGTL) and phosphorylation of hormone-sensitive lipase (HSL) [93,94]. The study of Chang et al. [94] demonstrated that increasing lipolysis associated with decreasing lipogenesis could be induced by activation of sirtuin 1 (Sirt1)/AMPK pathway [94].

## 5. Conclusions

This study highlighted the beneficial effect of the marine microalga *T. lutea*, as a dietary supplement, in the prevention of metabolic syndrome and obesity in Wistar rats. Metabolic disturbances associated with obesity were induced by a hyperlipidic diet and fructose-rich drinking water. Our results showed that *T. lutea* has an effective potential to reduce hyperglycemia, hypertriglyceridemia by restoring HDL level, hyperleptinemia and an excess of liver lipid levels, body weight and fat mass. Thus, the observed effects could be assigned to the bioactive molecules such as n-3 PUFAs, fucoxanthin, phytosterols, fiber, etc.) and their potential synergy within the biomass of *T. lutea*. However, this study did not determine the specific effect of each biomolecule from *T. lutea* biomass on physiological parameters involved in obesity and metabolic syndrome. The bioactivity of *T. lutea* purified extracts on inflammation, insulin resistance, and lipotoxicity should be further explored.

## Figures and Tables

**Table 1:** Effects of *T. lutea* on plasma biochemical parameters.

CTRL	CTRL	HF	HF-Tiso
**Plasma biochemical parameters**			
ASAT (UI/L)	58.72 ± 6.22	51.80 *** ± 7.83	45.09 ***^†^ ± 5.39
ALAT (UI/L)	44.46 ± 4.57	52.10 *** ± 4.10	52.00 *** ± 6.79
ASAT/ALAT	1.36 ± 0.11	1.24 *** ± 0.17	0.87 ***^†^ ± 0.09
Glucose (mmol/L)	9.17 ± 1.48	10.01 ***^†^ ± 0.58	7.73 ***^†^ ± 0.10
Insulin (µUI/L)	37.68 ± 8.26	89.17 *** ± 12.95	64.41 ***^†^ ± 11.69
Leptin (ng/mL)	2.02 ± 0.52	3.87 *** ± 0.53	2.17 ^†^ ± 0.30
HOMA-IR	0.97 ± 0.34	2.80 *** ± 0.57	1.30 ***^†^ ± 0.33
TAG (mmol/L)	3.07 ± 0.84	6.85 *** ± 1.16	3.53 ^†^ ± 1.02
TC (mmol/L)	2.14 ± 0.36	2.72 *** ± 0.45	3.64 ***^†^ ± 0.35
HDL-C (mmol/L)	2.03 ± 0.27	1.72 *** ± 0.20	2.83 ***^†^ ± 0.26
LDL-C (mmol/L)	0.44 ± 0.12	0.96 *** ± 0.21	0.86 *** ± 0.19
HDL/LDL	2.65 ± 0.31	1.62 *** ± 0.39	2.73 ^†^ ± 0.44
AIP	0.32 ± 0.08	0.51 *** ± 0.13	0.18 ***^†^ ± 0.06

AIP, atherogenic index of plasma; ALAT, alanine amino-transferase; ASAT, aspartate amino-transferase; CTRL, standard diet; HDL-C, high-density lipoprotein cholesterol; HF, high-fat diet; HF-Tiso, high-fat diet supplemented with 12% of *T. lutea*; HOMA-IR, homeostasis model assessment of insulin resistance; LDL-C, low-density lipoprotein cholesterol; TAG, triacylglycerols; TC, total cholesterol. Values are means (*n* = 6), and statistical significance was determined using ANOVA with post hoc Fisher’s test. Mean values associated with *** were significantly different from those of the CTRL group (*p* < 0.001), and mean values with ^†^ were significantly different from those of the HF group (*p* < 0.05).

## Data Availability

Not applicable.

## Supplementary Materials

The following are available online at https://www.mdpi.com/2072-6643/13/2/430/s1, Figure S1: comparison of food efficiency between the different experimental groups, Table S1: biochemical composition of standard, high-fat diets, and freeze-dried *T. lutea* biomass, Table S2: fatty acid composition of diets, Table S3: pigment and sterol composition, antioxidant activity and in vitro digestibility of freeze-dried *T. lutea* biomass.
