# Supplementary material for: The Marine Microalga, Tisochrysis lutea, Protects against Metabolic Disorders Associated with Metabolic Syndrome and Obesity
Source: Nutrients. 2021 Jan 28;13(2):430. doi: 10.3390/nu13020430 (PMC7911999; doi:10.3390/nu13020430)
Supplement: Supplementary file 1 [file nutrients-13-00430-s001.pdf]

Supplementary materials

Table S1. Biochemical composition of standard, high fat diets and freeze-dried *T. lutea* biomass

	CTRL	HF	Tiso	
Nutrients (g/100g)	Data*	Data*	Mean	SD
Proteins	16	19	38.4	0.36
Carbohydrates	60	32	8.12	0.48
Lipids	4	36	37	
Mineral and vitamin mixture	5	5	-	
TDF†	4	-	11.17	8.77
IDF‡	-	-	6.29	5.90
SDF‡	-	-	6.06	3.21
Humidity	11	8	5.70	0.13
Ash	-	-	13.7	0.62

CTRL, standard diet; HF, high fat diet; IDF, insoluble dietary fiber; TDF, total dietary fiber; SDF, soluble dietary fiber; Tiso, freeze-dried of *T. lutea*; -, not analysed. For CTRL diet, the mineral mixture provides the following amounts in mg/kg of diet: C, 7300; Cl, 4000; Cu, 16; Fe, 270; K, 6000; Mg, 1600; Mn, 70; Na, 2500; P, 5500; Zn, 55. The vitamin mixture of CTRL diet provides the following amounts in mg/kg of diet: biotin, 0.04; cholin, 1600; folic acid, 0.5; niacin, 75; vitamin A, 7500; vitamin B1, 7; vitamin B2, 6.5; vitamin B5, 16.5; vitamin B6, 2.6; vitamin B12, 0.02; vitamin D3, 1000; vitamin E, 30; vitamin K3, 2.5. For HF diet the mineral mixture provides the following amounts in mg/kg of diet: Ca, 3000; Na, 4100; P, 5000. Results are represented as mean values ± SD, n = 3 for proteins, carbohydrates, humidity and ash of Tiso biomass. * Data of biochemical composition of control and high fat diets have been furnished by SAFE; † Mean values (n = 2) are shown for TDF of Tiso biomass. ‡ Values (n = 1) are shown for IDF and SDF of Tiso biomass.

Table S2. Fatty acid composition of diets

	CTRL (% molar)	HF (% molar)	Tiso (g/100g)	
Fatty acid	Mean	Mean	Mean	SD
4:0	-	1.49	ND	
8:0	-	1.20	ND	
10:0	-	3.07	ND	
12:0	-	3.95	ND	
14:0	0.63	13.16	2.89	
14:1	-	-	0.05	
15:0	-	-	0.03	
16:0	15.91	32.87	2.01	
16:1n-7	0.73	-	0.96	
16:2n-6	-	-	ND	
16:3n-3	-	-	ND	
16:3n-6	-	-	0.07	
16:4n-1	-	-	0.02	
16:4n-6	-	-	0.06	
18:0	2.00	13.32	0.13	
18:1n-7	-	-	0.17	
18:1n-9	21.44	16.92	2.45	
18:2n-6	49.84	5.44	1.54	
18:3n-3	4.23	-	1.76	
18:3n-6	-	-	0.1	
18:4n-3	1.28	-	1.73	
20:0	-	-	0.02	
20:2n-6	-	-	0.13	
20:3n-3	-	-	0.03	
20:4n-3	-	-	0.03	
20:4 n-6	-	-	0.04	
20:5 n-3	0.66	-	0.07	
22:0	-	-	0.29	
22:1	-	-	0.1	
22:5n-3	0.91	-	0.03	
22:5n-6	-	-	0.02	
22:6n-3	1.03	-	0.21	
24:0	-	-	1.41	
Others	1.24	8.58	0.02	
Total SFA	18.54	69.06	0.09	
Total MUFA	22.17	16.92	5.22	
Total PUFA	58.05	5.44	3.79	
Total n-3 PUFA	8.11	-	7.37	
Total n-6 PUFA	49.94	5.46	5.27	
n-6 PUFA/ n-3 PUFA	0.16	-	2.08	
Total fatty acids	98.76	91.42	2.53	

CTRL, standard diet; HF, high fat diet; ND, not detected; Tiso, freeze-dried of *T. lutea*; -, not analysed. Results are represented as mean values ± SD, n = 3 for Tiso biomass. Mean values from CTRL and HF diets (n = 2) were similar, no statistical analysis were carried out, no SD mentioned.

Table S3. Pigment and sterol composition, antioxidant activity and *in vitro* digestibility of freeze-dried *T. lutea*

	**Tiso**	
Pigments (g/100g)	Mean	SD
Total chlorophyll	0.76	$0.03\ 10^{-1}$
Chlorophyll-*a*	1.30	$0.04\ 10^{-1}$
Chlorophyll-*c*	0.98	$0.02\ 10^{-1}$
Total carotenoids	0.76	$0.03\ 10^{-1}$
Carotenoids (g/100g)		
Fucoxanthin	$4.9\ 10^{-1}$	
4k-hex-fucoxanthin	$1.61\ 10^{-1}$	
Diadinoxanthin	$0.13\ 10^{-1}$	
β-carotene	$0.29\ 10^{-1}$	
Others	$0.69\ 10^{-1}$	
Sterols (g/100g)		
Cholesterol	$1.37\ 10^{-3}$	
Brassicasterol	$9.04\ 10^{-1}$	
24-Metilcholesterol	$5.5\ 10^{-3}$	
Campesterol	$8.24\ 10^{-3}$	
Stigmasterol	$2.11\ 10^{-1}$	
Fucosterol	$2.27\ 10^{-1}$	
Others	$1.51\ 10^{-2}$	
Total sterols	1.374	
Antioxidant activity*		
Antiradical activity (I_{50})† (DPPH in mg biomass/ml extract)‡	1.85	
Antioxydant activity ORAC (µmol TE/mg biomass)§	73.07	
In vitro digestibility (% dry matter)	75	2.90

DPPH, 2, 2-diphenyl-1-picrylhydrazyl; ORAC, Oxygen-radical absorbance capacity; TE, Trolox equivalent; Tiso, freeze-dried of *T. lutea*. Results are represented as mean values ± SD, n = 3, excepted for sterol composition (n = 1). * Antioxidant activity was measured from extracts of Tiso in 90% acetone. † I_{50}: concentration of the biomass/mL extract decreasing the absorbance of the DPPH solution by 50%. ‡ DPPH is a stable radical that can be reduced by reaction with an antiradical hydrogen-donor compound. § Trolox: water-soluble vitamin E analogue. 1 ORAC unit equals the net protection produced by 1 µM Trolox.

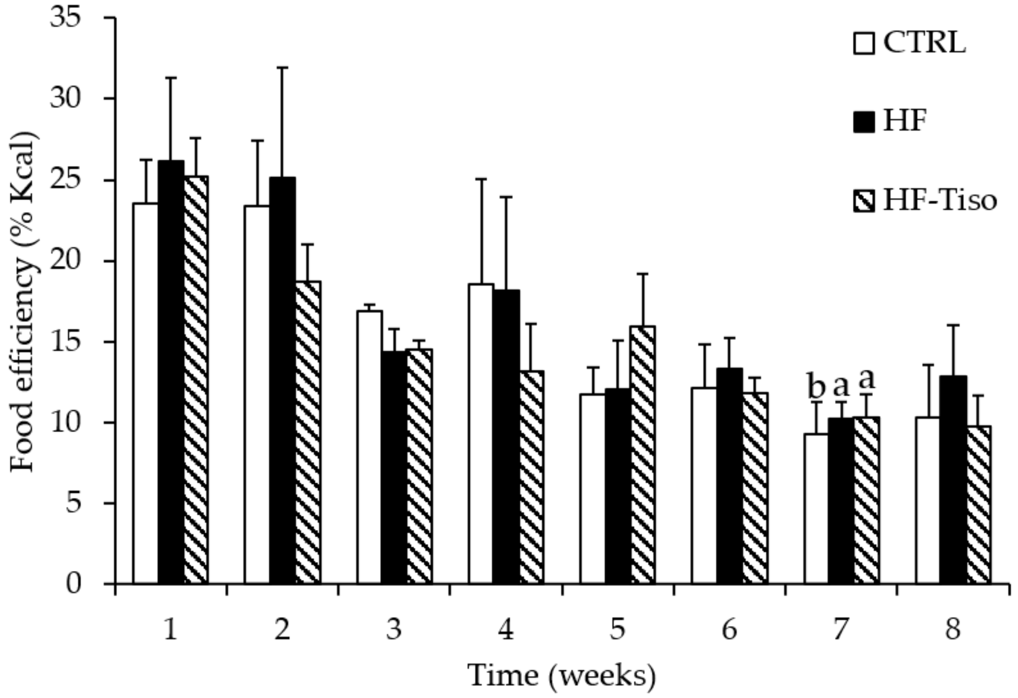

Figure S1. Comparaison of food efficiency between the different experimental groups. CTRL, control group; HF, high fat group; HF-Tiso, high fat group supplemented with *T. lutea*. Values are means (n = 6), with standard deviations represented by vertical bars. Statistical significance was determined using ANOVA with post-hoc Fisher's test and means associated with letters indicate significative difference at $p < 0.01$ with a > b.
